# Noninvasive real-time characterization of non-melanoma skin cancers with handheld optoacoustic probes

**DOI:** 10.1016/j.pacs.2017.05.003

**Published:** 2017-06-04

**Authors:** Amalina Binte Ebrahim Attia, Sai Yee Chuah, Daniel Razansky, Chris Jun Hui Ho, Pinky Malempati, U.S. Dinish, Renzhe Bi, Chit Yaw Fu, Steven J. Ford, Joyce Siong-See Lee, Melissa Wee Ping Tan, Malini Olivo, Steven Tien Guan Thng

**Affiliations:** a Bio-optical Imaging Group, Singapore Bioimaging Consortium, A*STAR, Singapore; b National Skin Centre, Singapore; c Institute for Biological and Medical Imaging, Technical University of Munich and Helmholtz Center Munich, Germany; d iThera Medical GmbH, Munich, Germany

**Keywords:** Photoacoustic imaging, Non-melanoma skin cancer, Tumor vasculature, 3D mapping, Histology

## Abstract

Currently, imaging technologies that enable dermsurgeons to visualize non-melanoma skin cancers (NMSC) *in vivo* preoperatively are lacking, resulting in excessive or incomplete removal. Multispectral optoacoustic tomography (MSOT) is a volumetric imaging tool to differentiate tissue chromophores and exogenous contrast agents, based on differences in their spectral signatures and used for high-resolution imaging of functional and molecular contrast at centimeter scale depth. We performed MSOT imaging with two- and three-dimensional handheld scanners on 21 Asian patients with NMSC. The tumors and their oxygenation parameters could be distinguished from normal skin endogenously. The lesion dimensions and depths were extracted from the spectral melanin component with three-dimensional spatial resolution up to 80 μm. The intraclass correlation coefficient correlating tumor dimension measurements between MSOT and *ex vivo* histology of excised tumors, showed good correlation. Real-time 3D imaging was found to provide information on lesion morphology and its underlying neovasculature, indicators of the tumor’s aggressiveness.

## 1 Introduction

Non-melanoma skin cancers (NMSC), the most common of which are basal cell carcinomas (BCC) and squamous cell carcinomas (SCC), is increasing worldwide [1] with low mortality rates [2]. However, NMSC can exert a considerable economic load to health providers and instigate morbidity since most NMSCs visibly appear on head, neck and face regions [3]. While the risk of metastasis associated with NMSC is rare [4], local invasion of the NMSC lesions can transpire three-dimensionally, destroying immediate structural tissues. These invasive NMSC lesions are either a result from treatment delay or the intrinsic characteristic of the lesion itself [4]. Furthermore, NMSC patients are at risk of developing a subsequent NMSC and non-cutaneous malignancy by ten-fold in contrast to non-patients [5]. The gold standard treatment for these skin cancers is surgical excision with post-operative histological and margin assessment or Mohs’ micrographic surgery (MMS). However, some forms of NMSC may have extensive subclinical involvement with an increased risk of incomplete clearance. Though MMS would be the best option in these patients, there are risks of excess tissue damage if excised or prolonged surgery time due to the need for multiple resections with frozen sections if treated with MMS. The development of new skin imaging techniques providing non-invasive visualization without the need for excisional skin biopsy is an emerging trend in dermatology. With these skin imaging techniques that allow better in vivo visualization of skin cancers, surgeons will be able to plan and individualize surgery for patients with the aim to reduce damage to normal tissue, surgical time and better clearance. However, continuous efforts are needed to develop an imaging system that enables more accurate skin cancer dimensions measurements. The dermsurgeons can greatly benefit from the information provided by these imaging modalities to plan and individualize the surgery for patients more effectively, with the aim to reduce tissue damage, surgical time and better clearance thus positively impacting the long-term outcome of these procedures.

Traditional non-invasive imaging modalities operated via a convenient handheld approach employed in the field of skin diagnostics are reflectance confocal microscopy (RCM), optical coherence tomography (OCT) and ultrasonography [6]. However, their diagnostic capacity is hampered by the lack of spatial resolution, penetration depth or contrast. The penetration depth of optical microscopy and OCT is limited to the first millimeter as a result of tremendous photon scattering in the skin [7] while other clinical imaging modalities lack the functional contrast in discriminating different tissue chromophores. For example, hypoechoic skin lesions cannot be distinguished from other hypoechoic areas such as fat in ultrasound [6]. Multispectral Optoacoustic Tomography (MSOT) is a non-invasive, high resolution, intrinsic or contrast-enhanced imaging technique which can provide functional and metabolic information at greater depths. Since MSOT detects ultrasound waves produced by absorption of pulsed light, it is capable of visualizing the diverse optical contrast of tissues without suffering resolution degradation due to photon scattering. Endogenous chromophoric substances that can be detected by MSOT include melanin, oxy- and deoxy-hemoglobin, water and lipids. As a result, functional information such as saturation of oxygen can be calculated from total and fractional hemoglobin [8], which is particularly useful in studying tumor angiogenesis. Information about melanin has been demonstrated to be highly valuable in studying melanoma in lymph nodes [9]. The ability of MSOT to resolve melanin and hemoglobin spectra makes it par ticularly useful in the study of skin diseases [10]. Ford et al. has demonstrated that MSOT was able to resolve small vasculature and other anatomical features with spatial resolution of 60 μm as deep as 4.7 mm under the surface of the skin [11]. Custom-made handheld scanners 2D and 3D MSOT handheld probes have been developed with the aim of clinical translation, and it has demonstrated the great potential for the clinical use of these probes [12], where the imaging performance of 2D and 3D probes in melanoma metastasis in a mouse brain model has been studied *in vivo*.

In the event that a lesion has been determined as cancerous and needs to be excised, it is important to determine the tumor dimensions accurately. Therefore, volumetric information from deep tissue layers on the melanin concentration and vascularization would enable preoperative mapping of the skin cancer and help the surgeons perform the excision more precisely, which may translate to lower relapse rates. Preoperative mapping of the lesion can additionally provide lesion vascular features which can determine the type of the BCC [13]. The goal of our study was to determine whether MSOT imaging could be exploited for determining tumor dimensions non-invasively and to study the vasculature features of skin tumors. We imaged the skin tumors *in vivo* preoperatively and resolved their existing endogenous chromophores (melanin, hemoglobin) to give a spatial map of the absorbers. The skin tumors’ dimensions (length and depth) were compared to their corresponding histological measurements following excision or MMS.

## 2 Material and methods

### 2.1 Study population

Twenty-one Asian patients fulfilling the inclusion criteria − above 21 years, presented with lesions suspicious of NMSC, and scheduled for excision with margin or MMS were recruited within a period of 5 months. These lesions were imaged with MSOT prior to surgery. Patients who refuse surgery were excluded. Patients were imaged in compliance with respective institutional approvals and with written consent. Protocol was approved by the Domain Specific Review Board (DSRB) of National Health Group, Singapore (Ref No. 015/00462). Eleven patients were imaged using the MSOT 3D probe while the remaining patients were imaged with the 2D probe.

### 2.2 Histology

All the excised skin lesions were examined histologically using the standard stain with hematoxylin and eosin (H&E).

### 2.3 MSOT imaging

Trained research personnel acquired in vivo optoacoustic images of skin via the inVision 512-echo (iThera Medical GmbH, Munich, Germany) MSOT system coupled with customized 2D or 3D handheld probes. The 2D probe provided cross-sectional 2D images in the x-z or y-z planes (z being the depth dimension, x and y are the two lateral dimensions), with an in-plane spatial resolution of 150 μm and effective field-of-view (FOV) of 25 × 25 mm. The 3D handheld probe comprises of a spherical matrix array providing a nearly isotropic resolution of 80 μm within an effective volumetric FOV of 6 × 6 × 6 mm. The 2D and 3D probes are readily interchangeable within the same optoacoustic imaging platform. Optoacoustic responses from the imaged tissue were generated by wavelength-tunable optical parametric oscillator laser whose output beam is guided into fiber bundles integrated into the handheld probes. The laser provides excitation pulses with duration of 9 ns at selectable wavelength from 680 nm to 980 nm at a repetition rate of 10 Hz and per-pulse energy of 100 mJ at 730 nm. A real-time image preview window generated based on the backprojection algorithm was available during data acquisition. The light intensity was attenuated in both 2D and 3D probes to ensure compliance with ANSI limits on the maximum permissible exposure (MPE) of human skin to pulsed laser radiation [14]. MSOT images were acquired using both handheld imaging probes at multiple wavelengths – 700, 715, 730, 760, 780, 790, 800, 825, 850 and 900 nm – 3 frames were averaged per wavelength. Clear ultrasound gel was present between the skin and detector for better acoustic coupling prior to imaging.

### 2.4 MSOT image processing

Optoacoustic signals acquired via both probes were used to reconstruct images via the back-projection algorithm in the proprietary ViewMSOT software suite (ver3.7; iThera Medical). After reconstruction, spectral unmixing of reconstructed data (negative values were discarded) was performed for each pixel to differentiate the spectral signatures of tissue chromophores (Hb, HbO_2_, melanin) [8]. In this way, the tissue chromophores can be resolved spatially and quantitatively, and then overlaid in a pseudo-colour scheme to form an image representing spatial distribution of the different bio-chromophores in tissue. Real-time image display on a planar screen for the 3D probe was achieved through maximum intensity projections (MIP) in three orthogonal planes (*xy*, *xz*, and *yz*).

### 2.5 Tumor dimension measurements

Measurements in MSOT and histology were made at the deepest infiltration point of the tumors to obtain the maximum depth and length for both volumetric and cross-sectional imaging methods. Two of the patients were presented with more than one non-melanotic case and each skin lesion was treated as an individual case for comparison between histology and MSOT analysis. Dimensions of the tumor were then determined based on the distance between the non-baseline values of the melanin signal. Histopathology was done on the excised lesions taking into account the depth and longest dimension of the lesions in millimeters (authors JSSL and MWPT). These measurements were then compared with the MSOT measurements. Some limitations on the accuracy of the histology measurements included potential shrinkage of the specimens during processing and frozen section for specimens from MMS.

### 2.6 Statistical analysis

The intraclass correlation coefficient (ICC) was calculated to compare the tumor dimensions (length and depth) for the same tumors between MSOT imaging and histology. The formula used is $ICC=\frac{k(SC)-SS}{(k-1)SS}$, where *k* = 2 (annotation values from MSOT and histology), SC is the sum of squares between patients and SS is the sum of total squares. ICC values were calculated for the length and depth of tumors in both cross-sectional and volumetric MSOT measurements in comparison to the corresponding histological measurements.

## 3 Results

### 3.1 3D volumetric imaging of skin tumors

**Fig. 1:**
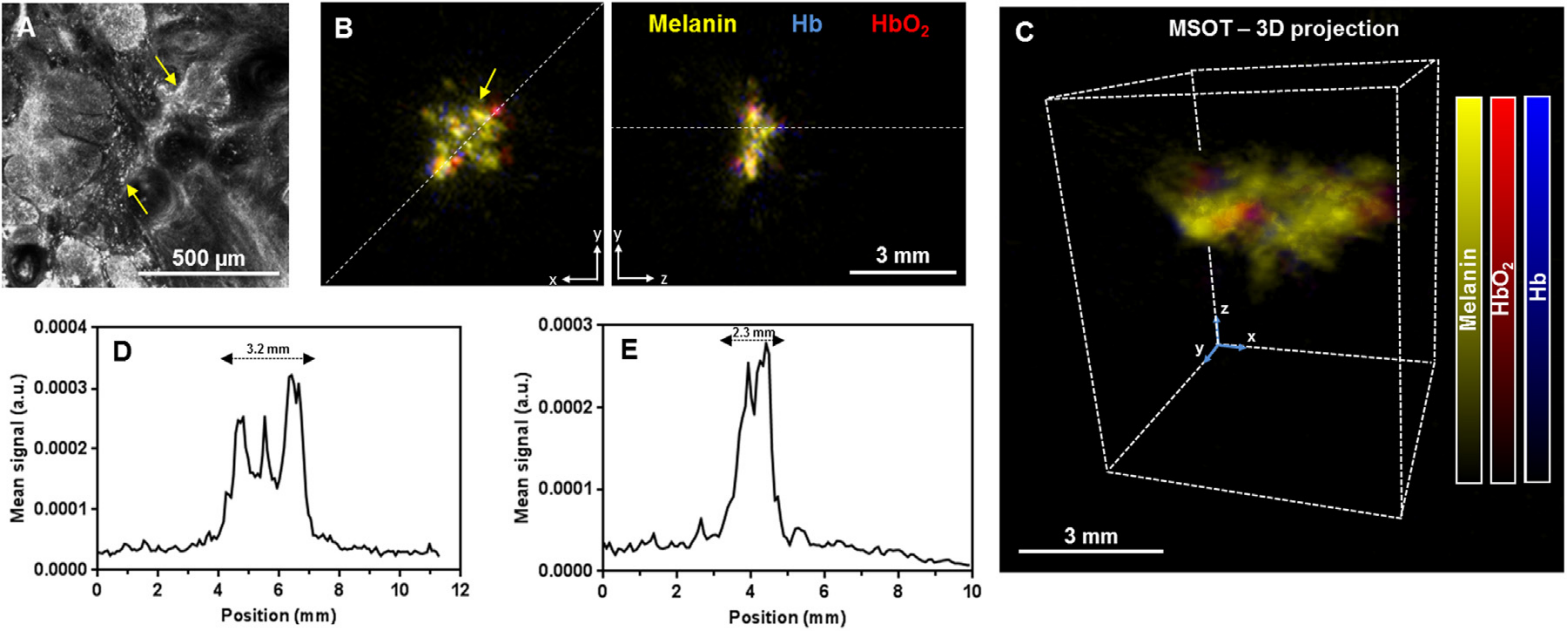
Single basal cell carcinoma imaged on the nose. (A) Reflectance confocal microscopy image of a representative BCC lesion showing uneven bright melanin signals (yellow arrows); (B) Maximum intensity projections of the volumetric MSOT images of the skin lesion in top (left) and cross-sectional (right) views; (C) Three-dimensional MSOT rendering of the BCC lesion shown in (B); MSOT melanin signal distribution along the (D) top and (E) cross-section axes as indicated by white lines in (B); a.u. − arbitrary units.

The 3D MSOT detector was utilized to image skin lesions in eleven patients without applying exogenous contrast. In one representative patient (Fig. 1), the reflectance confocal microscopy photograph of the skin lesion showed presence of tumor islands with peripheral palisading, clefting and uneven melanin bright pigmentation as denoted by the yellow arrows in Fig. 1A. These characteristics are attributes of BCC. Using the 3D handheld detector, we obtained orthogonal views of the BCC lesion rendering a map of melanin and hemoglobin signal distribution (Fig. 1B). Additionally, lateral views of the skin tumor and full 3D visualization could be obtained (Fig. 1C). The melanin signal distributions along the axes indicated by dotted white lines are shown in Fig. 1D (length) and Fig. 1E (depth). The dimensions of the tumor were then determined based on the distance between the non-baseline values of the melanin signal. The MSOT-measured dimensions in terms of depth and length were 2.3 and 3.2 mm, respectively, which were comparable to those obtained from histology (2.0 and 3.2 mm respectively, Fig. S1A). The other tumors imaged on other patients exhibited tumor dimensions similarity when derived from MSOT and histology.

### 3.2 Visualization of vasculature features in skin tumors

**Fig. 2:**
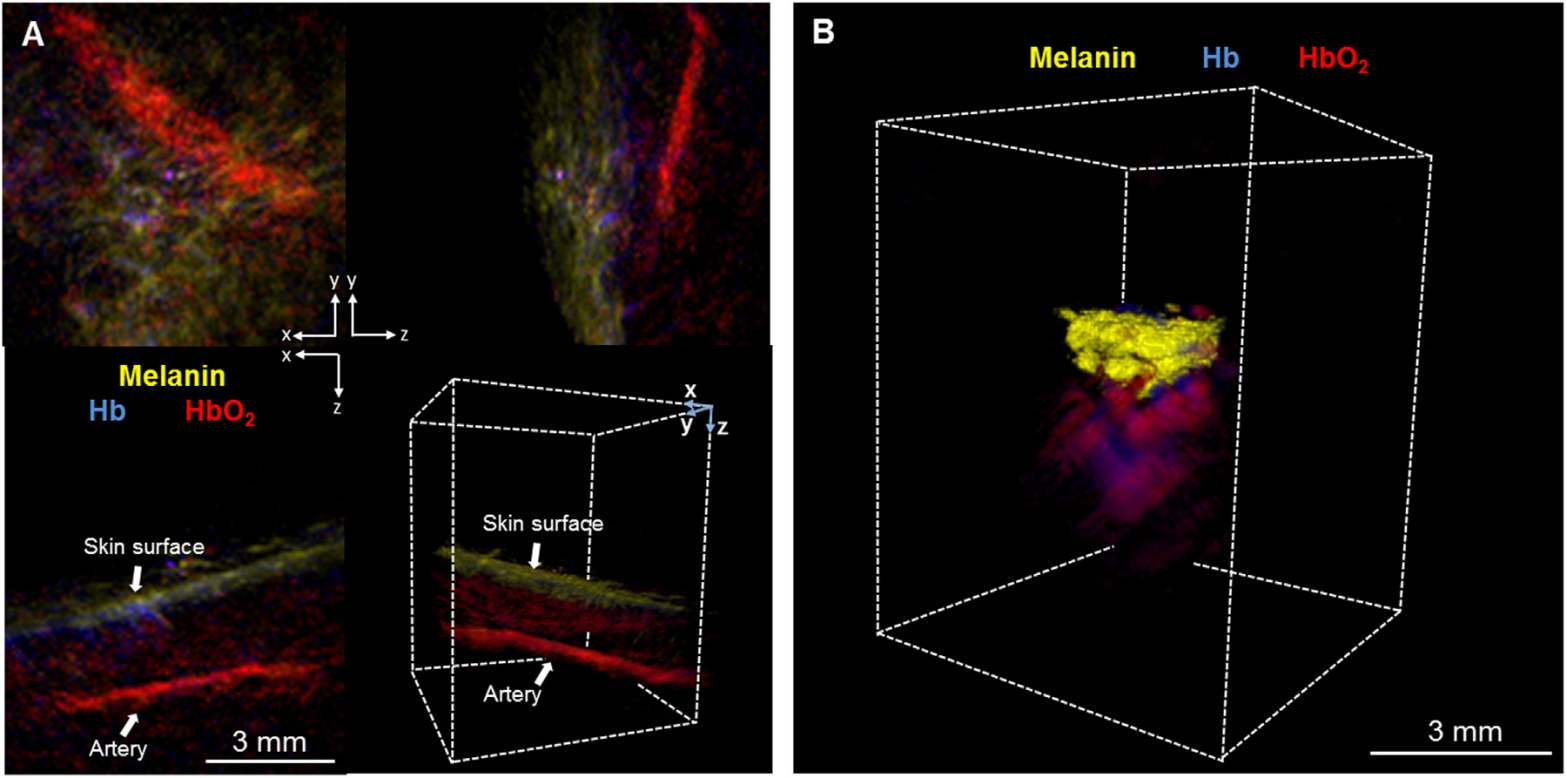
Optoacoustic imaging of deep-tissue vasculature (A) Orthogonal views and 3D rendering of MSOT images of a viral wart, with a depth estimated to be between 0.9 to 1.2 mm. A large artery, identified by characteristic oxy-hemoglobin (HbO_2_) absorption, was determined to be approximately 3.8 mm below the lesion. A rotating view of the MSOT 3D projection is in Video 1; (B) 3D MSOT rendering of a representative BCC lesion showing melanin (yellow), Hb (blue) and HbO_2_ (red) signals. Melanin signals were clustered at the top with strong hemoglobin signals underneath the BCC, showing deeper vasculature structures and the lesion’s aggressive subtype. A rotating view is in Video 2.

The 80 μm resolution conferred by the 3D handheld probe allowed for the visualization of tumor vasculature and oxygenation profiles. In one outlying case, the patient’s erythematous scaly plaque on the cheek was imaged with the 3D MSOT detector (Fig. 2A). Histological evaluation concluded it to be a viral wart (Fig. S1B), thus it was not included in the correlation between histological and MSOT measurements for NMSC. The MSOT images showed strong HbO_2_ signals beneath the lesion indicating the presence of prominent blood vessels, which is a common finding of a viral wart [15]. A single artery was observed at approximately 3.8 mm below the lesion. Oxy-hemoglobin signal was observed immediately below the lesion, delineating blood supply to the wart (Video 1). In another case where the patient was presented with nodular BCC on the nose, the MSOT images displayed clusters of melanin signals including intense HbO_2_ and Hb signals underneath the tumor, suggesting extensive vascularization of the tumor (Fig. 2B, Video 2). Tumor vascularity is recognized as a probable hallmark of aggressive BCC subtypes, which can determine the therapeutic regimen. This particular tumor was excised through MMS and, notably, the accumulative depth of the melanin and the hemoglobin signals corresponded to that from histological measurements (3.2 vs. 3.4 mm, histological and MSOT respectively).

### 3.3 Imaging of skin tumors with the cross-sectional (2D) probe

**Fig. 3:**
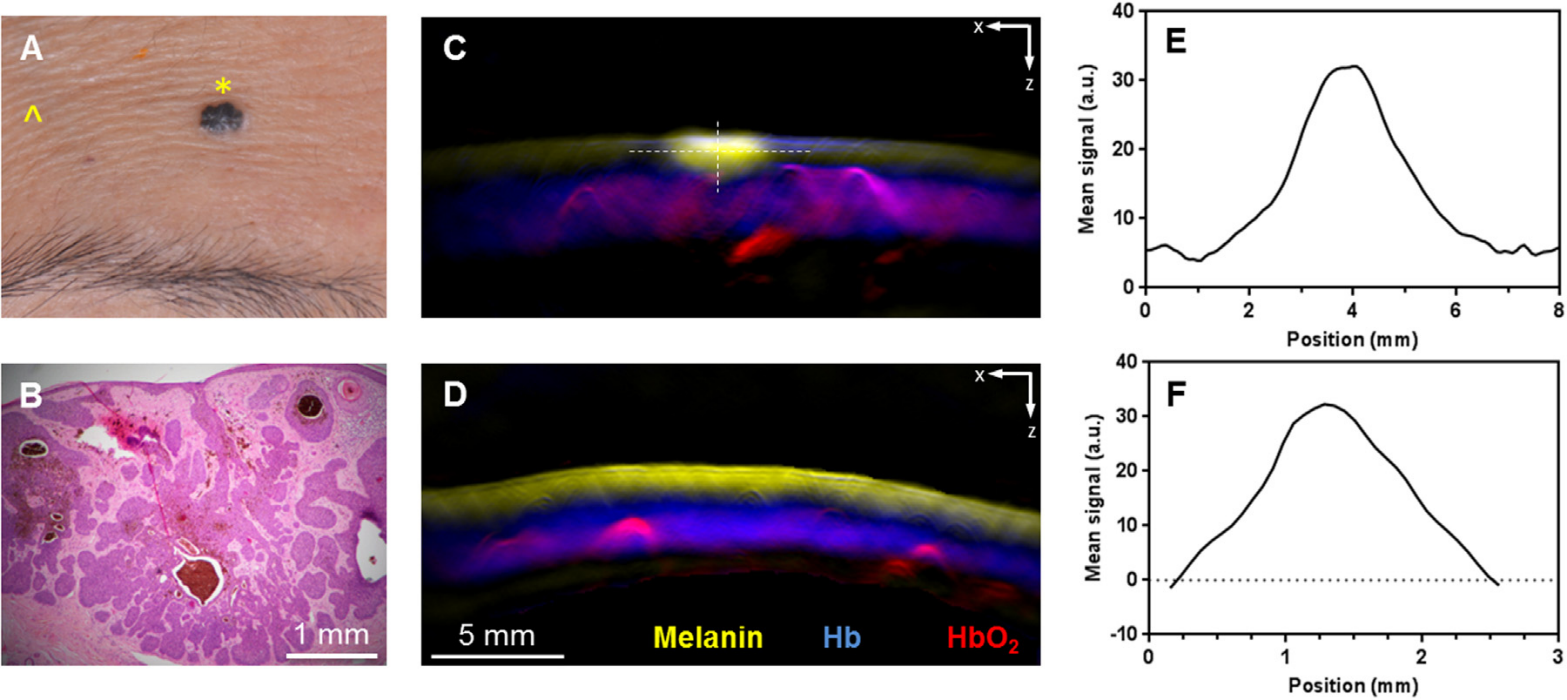
Optoacoustic imaging of basal cell carcinoma using 2D hand-held probe. (A) Clinical image of a representative BCC lesion on the forehead; (B) Histology confirmed the lesion to be BCC (×200 magnification); MSOT images acquired using the 2D probe of the (C) lesion as imaged at the yellow star in (A) and (D) normal skin as imaged at the yellow arrow in (A) indicating melanin, Hb and HbO_2_ signals; Melanin MSOT signal distribution along the (E) length and (F) depth dimension of the lesion, as indicated by the white lines in (C). a.u. − arbitrary units.

Ten patients were imaged with the 2D MSOT detector to obtain the tumor dimensions (length, depth) of 14 different skin lesions. Two of the patients had more than one lesion whereby one patient presented with two BCC lesions while the other presented with four BCC lesions near the hairline and scalp. A BCC on the forehead was diagnosed on one representative patient; shown in Fig. 3a. The skin tumor was then excised and sent for histology analysis (Fig. 3B). The histology showed proliferation of basal cells along the epidermal axis. The 2D MSOT image showed a bright melanin spot on the skin surface representing the BCC (Fig. 3C). Strong deoxy and oxy-hemoglobin signals were observed underneath the layer of melanin, indicating vasculature in the dermis layer of the skin. In contrast to normal skin (Fig. 3D), the melanin signals were evenly distributed on the uppermost layer. The melanin signal distribution provided MSOT-measured lesion dimensions of 2.5 and 1.2 mm in width (Fig. 3E) and depth (Fig. 3F) respectively, based on full width at half maximum. The distance between non-baseline values of melanin gave width and depth dimensions of 6.2 and 2.3 mm respectively. In comparison, the histological analysis has rendered 2.55 and 1.65 mm for the width and depth, respectively.

### 3.4 Correlation of tumor dimension MSOT measurements to histology

**Fig. 4:**
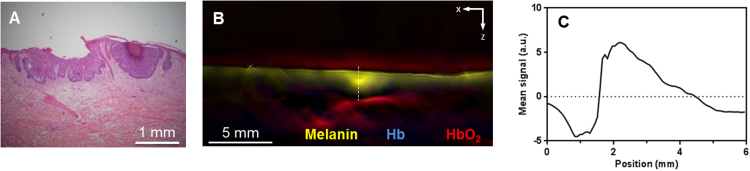
Optoacoustic imaging of superficial basal cell carcinoma using 2D hand-held probe. (A) Histology of a representative superficial BCC lesion on another patient’s back; (B) The corresponding MSOT image acquired using the 2D probe, showing a melanin signal at a larger depth than measured by histology; (C) Melanin MSOT signal distribution along the depth dimension of the superficial BCC in (A), as indicated by the white line on (B).). a.u. − arbitrary units.

The depth and length of the skin tumors were measured by histology and MSOT. The ICC values were used for a similarity analysis between the two modalities measuring the same tumor. Tumor depth and length measurements via MSOT imaging with the handheld 3D detectors compared to those from histological measurements exhibited a good correlation (ICC = 0.81 for depth and length), even though the measurements were performed with live tissues in MSOT versus dehydrated tissues in histology. The similar dimensions conferred by MSOT and histology indicated that pre-operative MSOT imaging is a worthwhile and reliable method to plan the excision surgery and to ensure a one-time surgery while minimizing the removal of healthy skin. However, tumor depth and length measurements via the handheld 2D MSOT detector in relation to histological measurements based on full width at half maximum and distance between zero-crossing points displayed poor correlation (ICC = 0.23 depth; ICC = 0.61 length for full width at half maximum) probably due to its vastly unisotropic resolution which gave overestimates of the length and depth. In one representative lesion on the back, the histology of the lesion (Fig. 4A) showed superficial BCC with shallow budding of tumor cells in the epidermis with no penetration into the dermis. The BCC lesion was found to be 0.4 mm deep in histology. In contrast, the MSOT image exhibited a strong melanin signal of more than 2 mm depth, most likely representing the BCC lesion (Fig. 4B and C). The overestimation of the lesion depth could be attributed to the highly unisotropic resolution conferred by the 2D handheld detector, indicating it may not be suitable for accurate characterization of superficial skin conditions.

## 4 Discussion

NMSC in particular BCC has been studied by other imaging techniques such as OCT and ultrasound [16], [17]. However, continuous efforts are needed to develop an imaging system that enable more accurate skin cancer dimensions measurements. Herein, MSOT was exploited to image the structural features of BCCs that serve advantages such as imaging at deeper tissue level while concurrently giving information about its tissue components and tumor dimensions. All the studied skin lesions had recognizable images on MSOT with both detectors, visualizing the shape and thickness of the lesions. The handheld probe design of the MSOT detectors permitted the imaging of BCCs on various common locations (forehead, nose, cheek, chin, arm, back and hairline). In addition, multispectral methodology of unmixing the optoacoustic signals permits the spatial differentiation of tissue absorbers in each lesion to visualize other parameters such as perfusion and blood oxygenation in the vicinity of the BCC lesion. These morphology patterns of the lesions vascularity as shown in MSOT would provide an additional dimension in studying the lesion. Aggressive types of skin cancers can involve deeper structures such as predominant deep blood vessels, which can be best detected when using the 3D handheld imaging approach. In the case shown in Fig. 2B particularly, the depth of the BCC which included its underlying vasculature reached beyond 3 mm which may be undetected by other imaging modalities such as OCT.

MSOT imaging of the lesions using the cross-sectional detector notably gave an overestimation of the BCC depths and limited information on the vasculature surrounding the lesion. In particular, the control skin on the forehead imaged by MSOT (Fig. 3D) exhibited melanin signals up to a depth of 1.4 mm with vasculature about 2.1 mm in thickness underneath. The skin (epidermis and dermis) thickness on the forehead may vary from 0.90 to 1.16 mm [18]. Due to the limitation in the resolution of the 2D probe, its visualization of the skin layers may not be discrete and hence the melanin signals were present on both the epidermis and dermis layers on the control skin. As the skin vasculature is also present in the MSOT control skin images, it is difficult to differentiate the healthy skin vasculature from the vessels feeding the lesion. The thickness of the lesion therefore may not be representative of the extent of the tissue in the depth dimension that has to be removed to prevent recurrence. Furthermore, there is a need to image several cross-sections of the tumors, in order to obtain the greatest depth and an overall view of the tumor.

## 5 Conclusions

Both the 2D and 3D MSOT handheld detectors offered structural and functional images of the skin tumors with well-resolved tissue chromophores. Yet, the unique real-time 3D imaging capacity provided by the volumetric handheld MSOT probe was found to be more suitable to image the skin cancer lesions, giving more accurate tumor dimensions compared to those from histology analysis, and better visualization of underlying deeper vasculature. Even though cross-sectional probe may in principle provide a greater imaging depth, it offered inadequate accuracy in determining the depth of melanin signals in the lesion. Despite the limited number of patients in this study, we were able to show that the volumetric MSOT imaging shows great promise in mapping and visualization of the skin cancer lesions, to be used as an assistive tool in guiding surgical interventions. Its noninvasive real-time imaging characteristics further make it an ideal modality for longitudinal monitoring of skin diseases and treatment responses.

## Conflicts of interest

SJF is an employee of iThera Medical, GmbH. A Research Collaboration Agreement free of financial interests was initiated between Singapore Bioimaging Consortium and iThera Medical, GmbH.

## Appendix A Supplementary data

Fig. S1 Histology images of representative skin lesions. (A) for BCC in Fig. 1 and (B) for viral wart in Fig. 2a.

Video 1 Rotating 3D projection of a viral wart.

Video 2 Rotating 3D projection of a BCC lesion with vasculature underneath.
