# Supplementary figures and images for: Noninvasive real-time characterization of non-melanoma skin cancers with handheld optoacoustic probes
Source: Photoacoustics. 2017 Jun 4;7:20–6. doi: 10.1016/j.pacs.2017.05.003 (PMC5473546; doi:10.1016/j.pacs.2017.05.003)

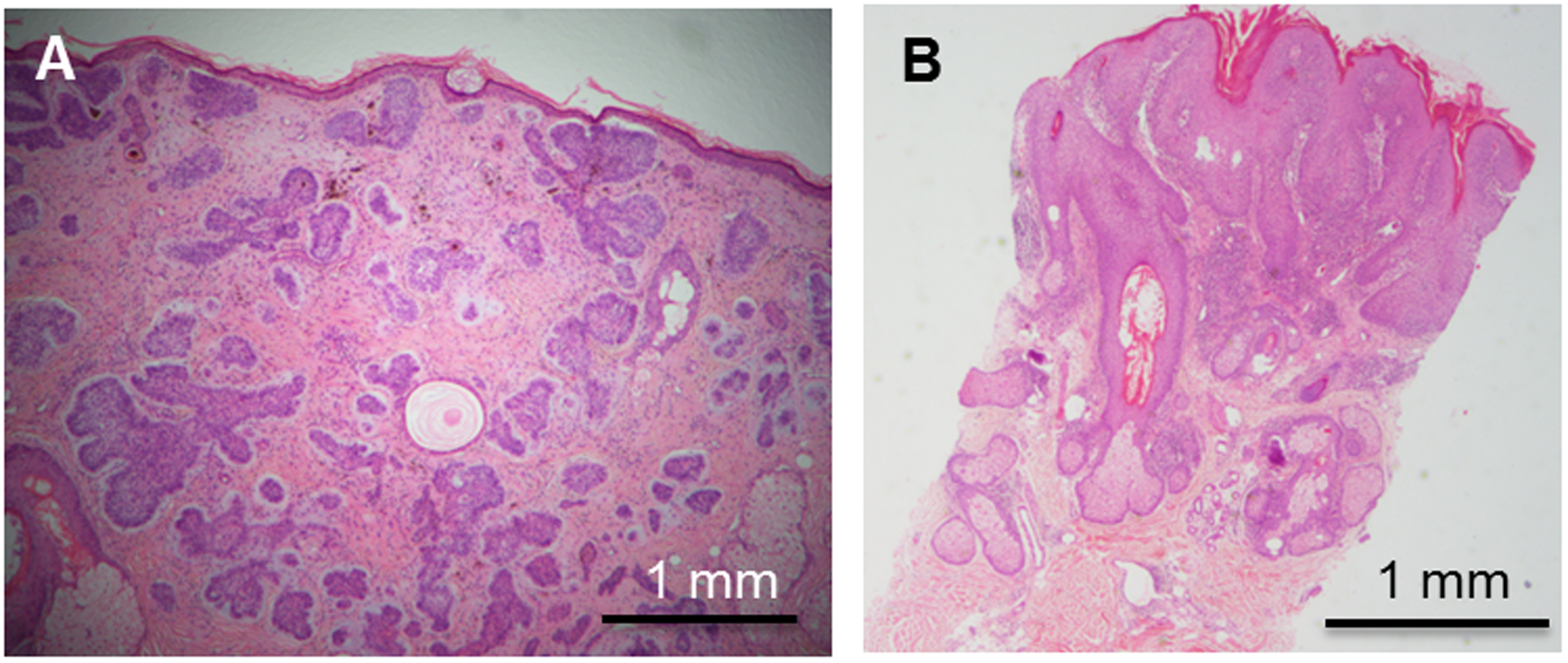

Supplement: Fig. S1 — Histology images of representative skin lesions. (A) for BCC in Fig. 1 and (B) for viral wart in Fig. 2a. [file mmc1.jpg]

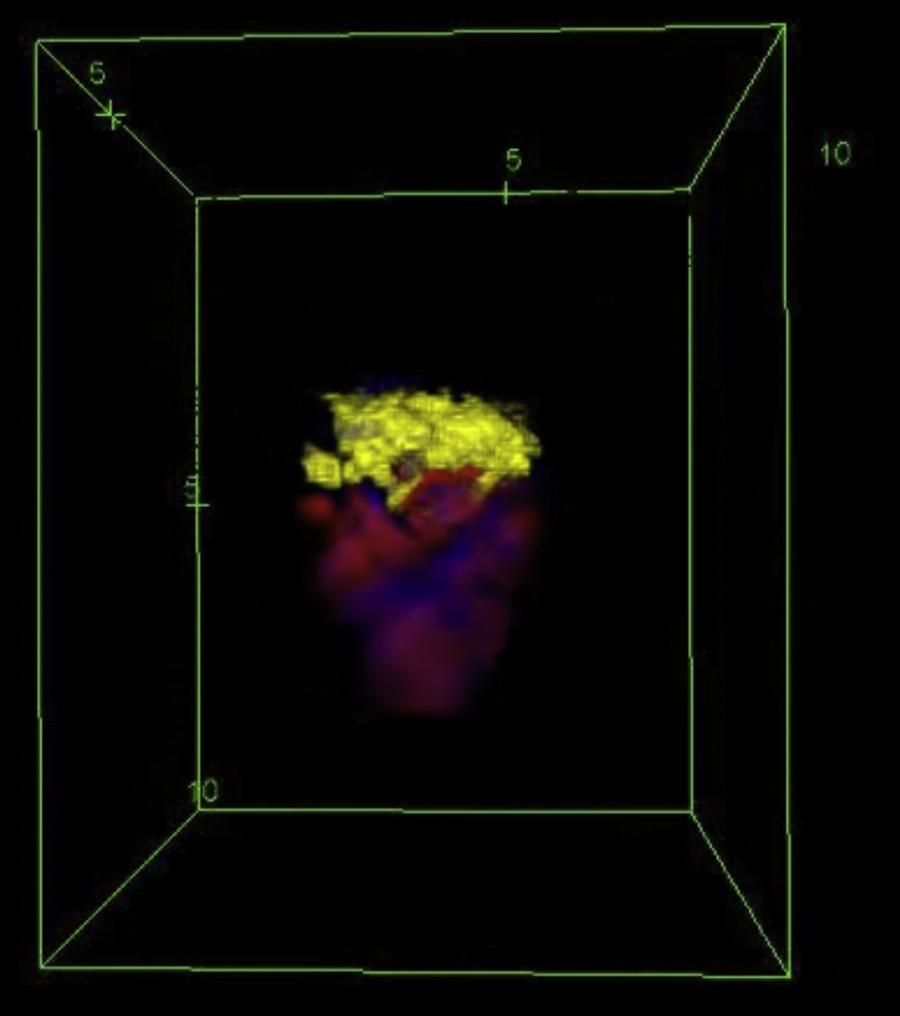

Supplement: Video 1 — Rotating 3D projection of a viral wart. [file mmc2.jpg]

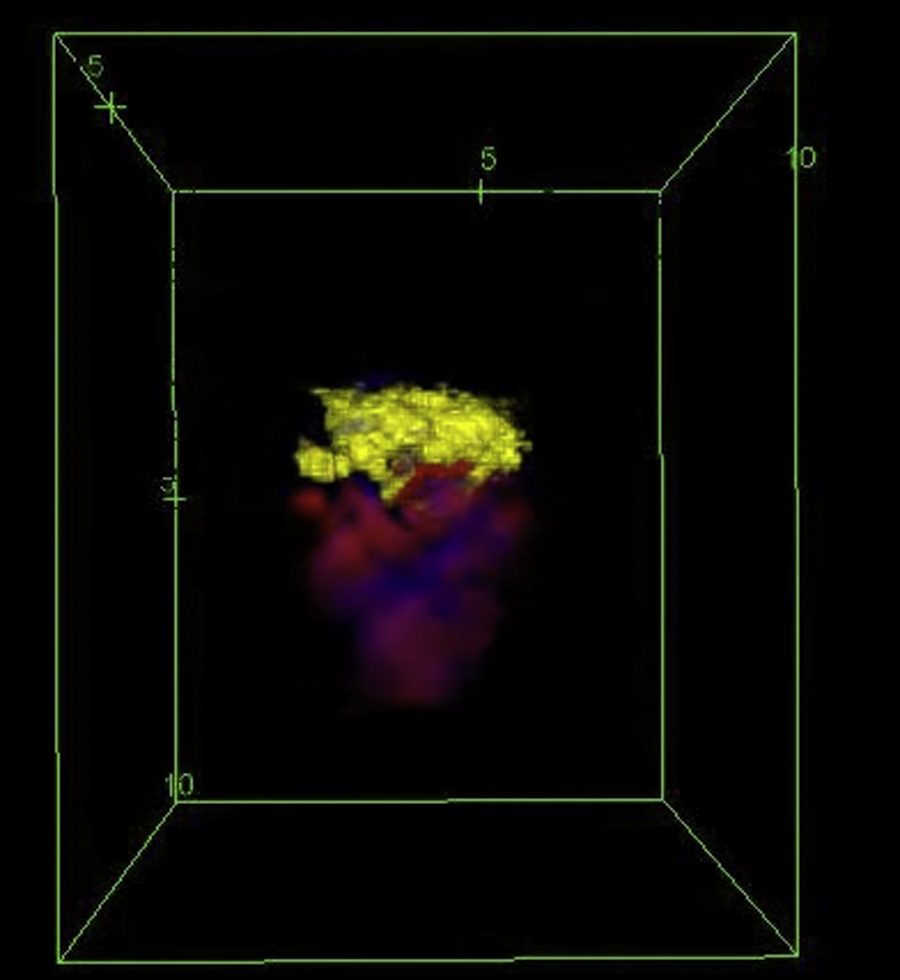

Supplement: Video 2 — Rotating 3D projection of a BCC lesion with vasculature underneath. [file mmc3.jpg]
